# Surgical debridement in long bone chronic osteomyelitis: is wide tumour-like resection necessary? evolving concepts and a review of cases in a tertiary bone infection unit

**DOI:** 10.1302/2633-1462.48.BJO-2023-0017.R1

**Published:** 2023-08-24

**Authors:** Mickhael B. Langit, Kae S. Tay, Hussain K. Al-Omar, Gavin Barlow, Joanna Bates, Cher B. Chuo, Ross Muir, Hemant Sharma

**Affiliations:** 1 Department of Orthopaedics, Hull University Teaching Hospitals NHS Trust, Hull, UK; 2 Department of Orthopedics, Philippine Orthopedic Center, Quezon City, Philippines; 3 Department of Infectious Diseases, Hull University Teaching Hospitals NHS Trust, Hull, UK; 4 Department of Radiology, Hull University Teaching Hospitals NHS Trust, Hull, UK; 5 Department of Plastic and Reconstructive Surgery, Hull University Teaching Hospitals NHS Trust, Hull, UK

**Keywords:** Osteomyelitis, Bone infection, chronic osteomyelitis, surgical debridement, long bone, antibiotics, bone infections, infection, wide resection, fracture-related infection, soft-tissue, bleeding

## Abstract

**Aims:**

The standard of wide tumour-like resection for chronic osteomyelitis (COM) has been challenged recently by adequate debridement. This paper reviews the evolution of surgical debridement for long bone COM, and presents the outcome of adequate debridement in a tertiary bone infection unit.

**Methods:**

We analyzed the retrospective record review from 2014 to 2020 of patients with long bone COM. All were managed by multidisciplinary infection team (MDT) protocol. Adequate debridement was employed for all cases, and no case of wide resection was included.

**Results:**

A total of 53 patients (54 bones) with median age of 45.5 years (interquartile range 31 to 55) and mean follow-up of 29 months (12 to 59) were included. In all, ten bones were Cierny-Mader type I, 39 were type III, and five were type IV. All patients were treated with single-staged management, except for one (planned two-stage stabilization). Positive microbial cultures grew in 75%. Overall, 46 cases (85%) had resolution of COM after index procedure, and 49 (90.7%) had resolution on last follow-up. Four patients (7%) underwent second surgical procedure and six patients (11%) had complications.

**Conclusion:**

We challenge the need for wide tumour-like resection in all cases of COM. Through detailed preoperative evaluation and planning with MDT approach, adequate debridement and local delivery of high concentration of antibiotic appears to provide comparable outcomes versus radical debridement.

Cite this article: *Bone Jt Open* 2023;4(8):643–651.

## Introduction

The current standard of management of long bone chronic osteomyelitis (COM) is guided by a number of principles: preoperative assessment, surgical debridement, microbial sampling, dead space management, soft-tissue coverage/reconstruction, systemic and local antibiotic therapy, and skeletal stabilization as required. ^1,2^

Surgical debridement remains the cornerstone of management of long bone COM. Successful treatment outcomes reported through time were anchored on “wide tumour-like resection” of infected bone.^2-5^ However, this does not come without causing structural instability and the need for extensive bone and soft-tissue reconstruction.^6^

In contrast, “adequate debridement” focuses on the debridement of grossly infected bone and soft-tissue while limiting resection of devitalized bone, hence preserving skeletal stability by avoiding segmental resection.^7^ This strategy, in combination with newer local antibiotic delivery systems and dead space management via a multidisciplinary approach, has been the strategy of the Hull University Teaching Hospitals Bone Infection Unit with good results.

This paper reviews evolving concepts in surgical debridement of long bone COM, from “wide, tumour-like” resection to “adequate debridement”, and reports the experience of our unit in the management of long bone COM from 2014 to 2020.

## Methods

This study was a single-centered retrospective review of patients with long bone COM treated at the Hull University Teaching Hospitals Bone Infection Unit from 2014 to 2020.

Records and imaging were electronically reviewed. Patients with long bone COM from fracture-related infection, hematogenous, and contiguous spread were included, with a minimum of 12 months' follow-up from the index procedure. COM was defined as history of symptoms for at least three months, with signs on clinical examination and imaging, and with presence of either sinus formation, pus or abscess during surgery, culture growth, or histological diagnosis.^8^ Exclusion criteria were wide segmental resection, lost to follow-up, or with incomplete records.

Demographics, anatomical site, aetiology, and comorbidities were recorded. Patients were classified based on Cierny-Mader (CM) type and host.^9^

All patients were managed via a multidisciplinary team (MDT) protocol. The MDT consists of orthopaedic surgeons specializing in bone infections, a bone and joint infectious diseases specialist, musculoskeletal radiologists, and plastic and reconstructive surgeons. The MDT protocol includes history taking, physical examination, and review of microbiologic history and imaging. All patients undergo standard radiography, CT scans assess the presence of sequestrum, and contrast MRI to determine extent of infection and plan the debridement.

Intraoperative findings and interventions, fixation, local antibiotic and carrier use, and need for a flap were recorded.

Microbiological data included number of samples, culture growth and types, number, and presence of resistant pathogens. Bacterial growth was positive if two or more phenotypically similar pathogens with similar (one susceptibility difference only) antimicrobial susceptibility patterns were isolated from deep tissue and/or fluid specimens. Superficial skin and sinus samples were not taken. Histology, where available, were also reviewed.

Recurrence of osteomyelitis (OM) was defined as the presence of signs and symptoms of infection, requiring further antimicrobial or surgical intervention. Resolution of OM as of last follow-up and complications, if any, were recorded. Our current follow-up protocol is face-to-face clinical review up to one year after surgery, then yearly telephonic review to identify late recurrences.

### Adequate debridement

Extent of adequate debridement is carried out based on imaging review. For CM type I cases, intramedullary nails are removed, canal reamed, and screw holes over drilled.

Access to the medullary canal is created for debridement of metaphyseal areas. A cloaca, if present, is extended to allow access as mentioned. Using multiple drill holes (2.7 mm), the channel is outlined and completed with an osteotome. Once with access, curettes and/or a burr are used, and reach is confirmed under fluoroscopy. At times, a dental mirror is useful to visualize difficult areas. This channel is created along the length of infected bone, and kept to 5 to 7 mm wide in long windows (> 10 cm), 7 to 10 mm in shorter windows (up to 10 cm), and 10 to 15 mm when muscle is used for biological dead space management.^10^ This process, compared to wide segmental resection, is tedious and time-consuming and risks an iatrogenic fracture. However, if done properly, extensive resection is avoided, maintaining skeletal stability.

The same is done for CM type II and III cases. The channel is created by connecting screw holes. Surfaces with biofilm are carefully decorticated using curettes, chisels, or burr. Previous screw tracts are over drilled. Less vital areas (i.e. minimal or no punctate bleeding in sclerotic bone) may be left behind to avoid destabilization, and are perforated with small drill holes to hopefully aid revascularization. Except for the paprika sign, bone viability is assessed using “point-of-care testing”, similar to Venter et al^11^ (bone colour, texture, etc).

For CM type IV cases, the above mentioned techniques were also employed, with the addition of skeletal stabilization. Wide, tumour-like resection was not done in cases included in this study.

High-volume saline (without antiseptic) was used for irrigation, often using pulsatile lavage for metaphyseal intramedullary surfaces and screw holes to dislodge debris. Calcium sulfate (Stimulan Biocomposites, UK) or calcium sulfate plus hydroxyapatite (CERAMENT-G; Bonesupport, Sweden) were used as local antibiotic carriers and dead space management. In some cases, antibiotic coated intramedullary nails were used, and one case used bioactive glass.

Primary closure of soft-tissue was done if possible. Otherwise, plastic surgeons cover the defect with local or free flaps. Patients initially receive individualized postoperative systemic intravenous and then oral antibiotics.

## Results

In all, 53 patients with 54 bones were included, with median age of 45.5 years (interquartile range 31 to 55), and 39 (74%) were male. The tibia was the most affected bone (50%). A total of 46 bones (85%) developed OM due to fracture-related infection, seven from hematogenous spread, and one from infection post arthroscopy (Table I). Follow-up was an average of 29 months (12 to 59). Table II shows the distribution per CM type. Culture growth was positive in 76% (Table III).

**Table I.** General characteristics.

**Variable**	**Data**
Patients, n	53
Bones, n	54
Median age, yrs (IQR)	45.5 (16 to 74)
Male sex, n (%)	39 (74)
**Bone involvement, n (%)**	
Tibia	27 (50)
Femur	10 (18)
Humerus	9 (17)
Fibula	5 (9)
Ulna	2 (4)
Radius	1 (2)
**Origin of OM, n (%)**	
Fracture-related	46 (85)
Hematogenous	7 (13)
Other (post-arthroscopy)	1 (2)

IQR, interquartile range; OM, osteomyelitis .

**Table II.** Cierny-Mader classification (type and host).

Cierny-Mader host type	Cierny-Mader host subtype	No.
I	IA	3
	IBs	4
	IBsl	3
Total, n (%)		10 (18.5)
II		0
Total, n (%)		0 (0)
III	IIIA	18
	IIIBl	3
	IIIBs	6
	IIIBsl	12
Total, n (%)		39 (72)
IV	IVA	2
	IVBs	1
	IVBsl	2
Total, n (%)		5 (9.5)

**Table III.** Bacterial isolates from procedures.

Variable	Data
Culture growth on index procedure, n (%)	n = 54
Yes	41 (76)
No	13 (24)
**Single vs polymicrobial growth on index procedure, n (%)**	**n = 41**
Single	29 (71)
Polymicrobial	12 (29)
**Bacteria on culture growth (single growth, index procedure), n**	**n = 29**
*Staphylococcus aureus*	19
*Enterobacter cloacae*	2
*Salmonella schwarzengrund*	1
Coagulase negative staphylococcus spp.	1
Meticillin-resistant *Staphylococcus epidermidis*	1
Group B haemolytic streptococci	1
*Staphylococcus mitis*	1
*Staphylococcus lugdunensis*	1
Anaerobes	1
*Pseudomonas* spp.	1
Bacteria on culture growth (multiple growth, index procedure)	n = 12
*Staphylococcus aureus + Enterococcus faecium*	2
*Staphylococcus aureus* + CNS	1
*Staphylococcus aureus* + MRSE	1
*Staphylococcus aureus + Morganella morgagni*	1
*Staphylococcus aureus* + MRSA	1
*Enterococcus faecalis + Bacillus licheniformis*	1
*Staphylococcus aureus + Klebsiella pneumonia + Enterococcus faecalis*	1
*Staphylococcus aureus* + group A β haemolytic streptococci + *Actinomyces neuii*	1
*Staphylococcus aureus + Staphylococcus aureus* strain 2	1
*Staphylococcus aureus + Staphylococcus epidermidis*	1
*Staphylococcus simulans + Staphylococcus caprae*	1
**Drug-resistant bacteria, n**	**n = 5**
*Enterobacter cloacae* (as single growth, first procedure)	1
MRSA (with methicillin-sensitive *S. aureus*, first procedure)	1
MRSE (on first procedure)	1
MRSE (with *S. aureus*, first procedure)	1
*S epidermidis* (on second procedure)	1

CNS, coagulase negative staphylococcus; MRSA, methicillin-resistant *Staphylococcus aureus*; MRSE, methicillin-resistant *Staphylococcus epidermidis*.

**Fig. 1:**
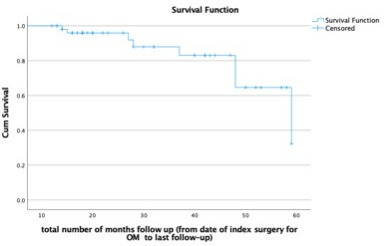
Kaplan-Meier curve of survival, until 60 months after surgery.

In all, 46 cases (85%) had no recurrence on last follow-up. Eight patients (15%) had recurrence, with median 236.5 days to presentation (IQR 76 to 845). Kaplan Meier survival curve (Figure 1) demonstrates this further. Four of the eight recurrences had second surgery, wherein three had resolution on last follow-up (Table IV). Of the four that did not undergo a second procedure, two had resolution on last follow-up. Out of 54 cases, the overall infection resolution rate on last follow-up was 90.7%.

**Table IV.** Recurrence of osteomyelitis after index surgery.

Age, yrs (sex)	Site and origin of OM	CM stage	Index surgery for OM	Days to recurrence	Initial microbiology	Recurrent microbiology	Second surgery for OM	Status as of last follow-up
73 (F)	Distal fibula ORIF lateral malleolus ~ 26 years prior	IIIBs	Arthrotomy + removal of plates and screws + application of local antibiotics (Stimulan)	83	*Pseudomonas* spp.	Nil	Re-debridement + application of local antibiotics (Cerament) + free AMT fascial flap + STSG	(+) resolution of OM; superficial flap ulceration
62 (M)	ORIF proximal tibia ~ 37 years prior	IIIBs	Debridement + removal of screws + application of local antibiotics (Cerament) + ALT free flap	791	*Staphylococcus aureus + Enterococcus faecalis*	*Staphylococcus aureus*	Re-debridement + application of local antibiotics (Stimulan) + gastrocnemius flap	(-) resolution of OM; occasional leak in proximal sinus, patient keen to manage conservatively
29 (M)	IMN femur ~ nine years prior	IBs	Removal of intramedullary nail + debridement + application of local antibiotics (Stimulan)	119	*Staphylococcus aureus + Enterococcus faecium*	N/A	None; advised repeat debridement but in interim symptoms became quiescent	(+) resolution of OM; Recurrence post-op with small sinus managed by daily dressing; sinus eventually closed by last follow-up
74 (M)	ORIF + surgery for infection of distal tibia ~ 25 years prior (debridement + flap + antibiotic beads)	IIIBsl	Drainage of abscess + debridement + application of bioglass (Bonalive)	182	*Staphylococcus aureus*+ MRSA	N/A	None (but was advised amputation due to poor vasculature of leg)	(-) resolution of OM
50 (F)	Proximal tibia circular frame fixation for refracture (previous ORIF distal tibia & fibula) six years prior	IIIBsl	Debridement + application of local antibiotic (Cerament)	291	No growth	N/A	None; noted recurrence from wound eight months post debridement; had second recurrence of symptoms but managed medically with antibiotics	(+) resolution of OM
49 (F)	ORIF tibial plateau four years prior	IIIBsl	Removal of plate + debridement + application of local antibiotics (Stimulan)	845	*Staphylococcus aureus*	N/A	None (poor patient follow-up)	(-) resolution of OM; with intermittent pus discharging from wound
34 (M)	Proximal tibia circular frame fixation of fracture	IIIA	Debridement + application of local antibiotics (Stimulan) + rotational flap	76	*Staphylococcus aureus*	*Staphylococcus epidermidis*	Re-debridement+ application of local antibiotic (Stimulan + Cerament) + medial gastrocnemius flap)	(+) resolution of OM
16 (F)	Proximal radius; hematogenous	IIIA	Debridement + application of local antibiotics (Stimulan)	442	No growth	No growth but on histopathology with 5 neutrophils per HPF (up to 3 HPF) noted consistent with OM	Excision of recurrent osteomyelitis + application of local antibiotics (Cerament)	(+) resolution of OM

CM, Cierny-Mader; N/A, not applicable; OM, osteomyelitis; ORIF, open reduction and internal fixation.

In all, 46 cases (85%) received a six-week regimen of antibiotics (one-week intravenous then five weeks oral). Two cases (4%) received less than six weeks, while six cases (11%) received 12 weeks. Vancomycin plus gentamicin or tobramycin were the local antibiotics in almost all cases (n = 44; 81.5%), with vancomycin plus tobramycin used most frequently (55%).

### Outcomes per cm type

A total of ten patients (18.5%) were CM type I: six previously with an intramedullary nail, three from hematogenous spread, and one after knee arthroscopy. Mean follow-up was 34.8 months. Removal of nail and reaming was done where applicable. Otherwise, access to the canal was created for debridement using the technique described. One case required a temporary circular external fixator after debridement (due to the extent of bone window created), and was stabilized with a hydrogel-coated intramedullary nail as a planned second-stage procedure.

One case had a sinus four months after debridement. However, during follow-up, the sinus has closed, and no further intervention was done. No patient from this group reported complications (Table IV).

While there were no CM type II OM reported, there were 39 cases of CM type III (72%). A total of 36 cases were fracture-related, three from hematogenous spread. Mean follow-up was 26 months. Implants were removed, cortical surfaces and screw holes debrided, with selected cases requiring access to medullary canal. In all, 28 cases used Stimulan, while seven used CERAMENT-G. Local antibiotics were not used in three cases; in one case, BonAlive (BonAlive Biomaterials, Finland) was used, in another case wherein the surgeon felt that local antibiotics were not required (frame extension to femur), and in one case, Stimulan was just introduced in our unit and the surgeon had reservations using it. Overall, 11 cases required a flap for soft-tissue coverage.

In all, seven CM type III cases had recurrence, wherein four had repeat surgery. Two cases had repeat debridement, local antibiotics, and a flap. The third case had a recurrent culture negative OM of the proximal radius, but was positive on histology. All three cases had resolution of symptoms on last follow-up. The fourth case had recurrence 791 days from debridement, and underwent repeat debridement, local antibiotics, and a flap. However, on follow-up, the patient had occasional discharge over the proximal tibia; repeat surgery was offered, but the patient chose conservative management (Table IV).

Three other cases of CM type III had recurrences but did not undergo repeat debridement. One case was advised amputation but refused. The other case had recurrence eight months after surgery, medically managed with antibiotics. The last case had intermittent discharge, but had poor follow-up and compliance.

In all, five cases had complications; three had sterile leak from local antibiotics, which eventually resolved, and two had sensory deficits (one around the knee, and one over the distribution of the superficial peroneal nerve).

There were five cases of CM type IV, four of which were fracture-related and one hematogenous spread. Mean follow-up was 42 months. One case was an infected segmental tibia fracture, and underwent removal of nail and reaming of canal, frame fixation, plus intramedullary antibiotic pellets. No wide resection was done. The second case was an infected subtrochanteric fracture nonunion, whereby an intramedullary nail was removed, reaming done, plus intramedullary antibiotic pellets. However, the limb was stable after nail removal and no wide resection was done, no fixation applied and nonunion eventually consolidated. The third case underwent debridement and exchange tibia nailing with a gentamicin-coated nail (Synthes Expert Tibia Nail PROtect; Synthes, Switzerland), and no wide resection was also done. These three cases had no recurrences nor complications on last follow-up.

**Fig. 2:**
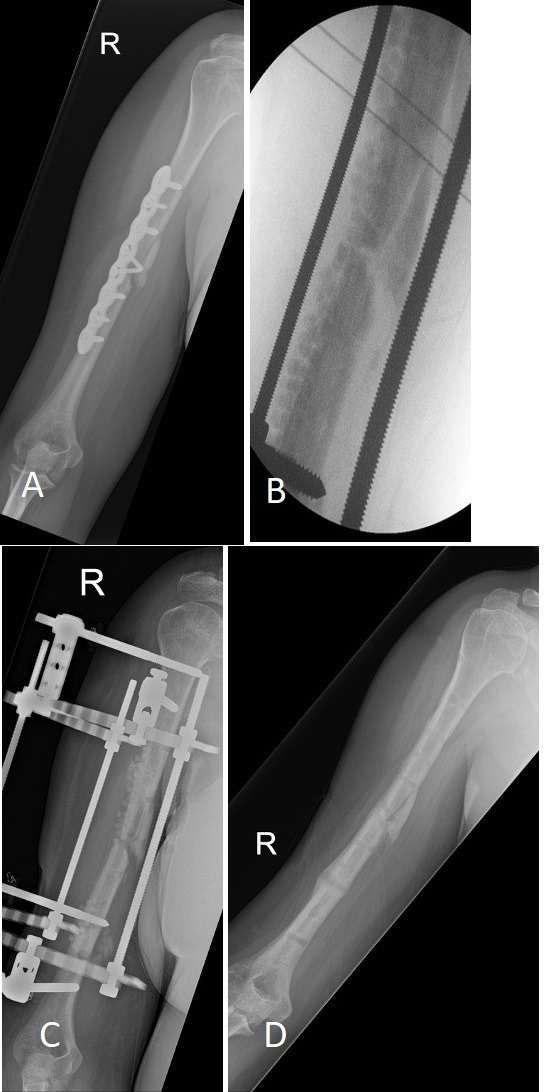
a) 39-year-old female type A host with infected nonunion of humerus fracture after open reduction and internal fixation. b) Intraoperative radiograph after plate removal, local antibiotics, and stabilization with frame. No wide resection was done. c) Interim radiograph showing good alignment and partial resorption of local antibiotics. d) Post removal of frame showing good union, no clinical signs and symptoms of recurrence of infection at 18 months after surgery.

The fourth case was an infected nonunion of a humerus fracture, which underwent removal of plate, debridement of cortex, over drilling of screw holes, and local antibiotics. The fracture was stabilized with a frame, and no wide resection was done. The fracture united with no recurrence of infection. However, the patient developed pin site OM due to the frame, and underwent debridement and local antibiotics. There was no recurrence of infection on last follow-up (Figure 2). The last case was a pathological femur fracture (hematogenous OM; intravenous drug user). The patient underwent debridement, reaming, local antibiotics, and frame fixation. However, the patient then required re-fixation with a frame ten months after first surgery after falling off a motorcycle. No recurrence of infection at two years and eight months after re-fixation.

### Discussion

Surgical debridement of COM has seen significant changes in practice through time. Avicenna in the ninth century highlighted the need for eradication of dead bone through “deep excavation.” If this is not enough, then amputation should be done.^12^ Significant developments through time included the use and eventual denouncement of cautery as treatment, and drainage and resection of dead bone.

Like Avicenna, the Orr treatment of pyogenic OM in 1936 emphasized total eradication of infected bone as much as possible.^13^ Total eradication was achieved through a generous incision, saucerization, and removal of all diseased bone. However, the Orr treatment did not come without complications. Prolonged immobilization in the cast (after significant bone resection) resulted in joint stiffness and contractures. In addition, immobilization did not promote circulation which further delayed healing. Hence, the treatment had various modifications including a longer and narrower corticotomy (to access the medullary canal) which provided sufficient bone stability for earlier mobilization.

Rowling et al^14^ in 1959 reported 58 cases of COM managed through a “sufficiently radical surgical attack” combined with soft-tissue reconstruction and antimicrobial therapy. In this series, the authors emphasized that “complete excision is essential,” and doing otherwise results in treatment failure. Similarly, many other authors presented their results anchored on “radical” debridement. McNally et al^3^ in 1993 reported 37 patients via a two-stage technique, emphasizing “radical debridement of all infected bone” and achieved union in 34. In “all cases, the excision of bone was radical with no attempt at a limited resection.” Bone with “abnormal appearance” were removed until bleeding was noted. Areas that did not bleed were re-examined and debrided to bleeding bone.

As such, wide tumour-like resection has been the prevalent paradigm in the management of OM for some decades now and, despite the difficulty in determining the limits of resection, is promoted by many authors, including Tiemann et al^15^ (2009), Forsberg et al^2^ (2011), Panteli et al^5^ (2016), and Barakat et al^1^ (2019).

Morelli et al^16^ reports that via the masquelet technique 91.1% of OM patients were in remission on last follow-up. Yalikun et al,^17^ on the other hand, reported an 11.4% recurrence of post-traumatic tibial OM treated with Ilizarov bone transport. These recurrence rates are comparable to the results of our series. However, excessive bone resection and the need for reconstruction comes with complications. Bone reconstruction usually is through distraction osteogenesis with external fixators or via masquelet technique.^6^ Liu et al^18^ reported that majority who underwent Ilizarov or monorail fixation suffered many complications during treatment. Common complications included pin site infection, axial deviation, soft-tissue incarceration, and delated union of docking site, and the most common major complication was joint stiffness. Morelli et al^16^ reviewed complications of the Masquelet technique. The technique requires two stages, which may not be suitable for certain populations. In addition, two-staged techniques increase morbidity and have cost implications to healthcare systems. The Masquelet technique for bone defects secondary to infection had a higher risk of complications, seen in almost half of cases (49.6%). Complications include superficial and deep infections, failure of either or both of the staged procedure, hardware failure, soft-tissue healing concerns, malalignment, joint stiffness, and refractures. In our study, two patients (4%) required surgery for complications. The most common complication was sterile leak from local antibiotics in three patients, which eventually resolved. Two reported mild sensory deficit after surgery. One had pin site OM secondary to external fixation of the humerus, and one required re-fixation of the femur after falling off a motorcycle.

Because of these, along with recent significant advancements in high concentration local antibiotic delivery and an MDT approach, adequate debridement has steadily challenged the need for wide, tumour-like resection in all cases of long bone COM. Adequate debridement is removal of enough bone to be able to debride all gross infection. Resection of devitalized bone is limited to gain access, which maintains primary stability of the bone. A channel is created as access for medullary debridement, and if a cloacae is present, it is extended to gain access as mentioned. In this technique, surgeons no longer look for punctate bleeding, or “paprika sign”, as a marker of healthy bone, meaning devitalized bone may be left behind.

Simpson et al^4^ in 2001 determined outcomes in patients with OM with varying debridement margins: wide resection with a margin greater than 5 mm, marginal resection less than 5 mm, and intralesional debridement. The wide resection group had no recurrence of OM versus 28% in the marginal excision group. In Simpson et al’s study, MRI was used to plan the extent of debridement but the extent of resection was decided during surgery. The paprika sign was used to determine viability of bone. In the marginal excision group, 50% of CM type B hosts had recurrence while there was no recurrence among type A hosts. Hence, the authors stated that they have shifted their practice towards marginal excision especially for type A hosts. Our study, with almost six of ten patients being type B hosts, reports a 15% recurrence rate compared to 28% in Simpson et al’s marginal excision group. With the use of local antibiotics, we suggest that adequate excision even in type B hosts may be sufficient.

Wide, tumour-like resection in search of the paprika sign was used to ensure local blood supply, hence delivery of systemic antibiotics. With the advent of dissolvable customized local antibiotic delivery systems with high minimum inhibitory concentration levels up to six weeks, the argument for wide resection is much weaker.

Single-staged management of OM is arguably the current gold standard when achievable. Successful outcomes of single-staged management of OM are made possible with advancements in local antibiotic delivery, dead space management, and soft-tissue coverage, a concept reported for a number of years now by McNally et al.^7^ This has been the experience of our unit, with an overall resolution rate of 90.7%. Segmental resections were more frequently performed in the early years, and with time has transitioned to most cases being treated with adequate debridement. Segmental resection still, however, has a role when indicated, such as in cases of grossly infected and unstable nonunion.

Overall, 17 studies showed a pooled non-recurrence rate of 90.9% via debridement and local antibiotics, similar to our unit’s results using this strategy.^19^ Ferguson et al^20^ used biodegradable calcium sulphate for dead space management and delivery of local antibiotics with a recurrence rate of 9.2% and eventual resolution rate of 97.9%. Our unit’s use of calcium sulfate had a recurrence rate of 15% and overall success rate of 90.7% yielded comparable results.

Prophylactic stabilization avoids fractures after resection of infected bone, particularly when less than 70% of the cortical volume remains.^10^ Chan et al^21^ reported approximately 15% of their patients with type II and III OM benefited from prophylactic fixation. In our technique, access to the medullary canal is done through a trough small enough to maintain structural stability. Fortunately, in our series, no patient sustained a pathological fracture.

In our study, eight patients presented with recurrence of symptoms after surgical debridement. Garcia Del Pozo et al^22^ determined risk factors for recurrence after treatment of long bone COM. Significant risk factors were duration of OM greater than three months, exposure of bone, and “treatment other than surgical debridement with muscular flap.” Interestingly, only one of our eight recurrences presented with bone exposure. On the other hand, seven of the eight recurrences in our series had a duration of greater than three months.

### Limitations

Our study has the inherent limitations of retrospective observational studies. There was no control group that underwent wide resection, which might have provided more insight on the benefits and disadvantages of adequate debridement. There is also the risk of selection bias, although in the same period of this study, our unit performed only four segmental resections for appropriate cases.

In conclusion, with the advent of local antibiotic delivery systems, the argument for wide, tumour-like resection during debridement of long bone COM is much weaker. Adequate debridement of COM achieves success through detailed preoperative evaluation and planning via MDT approach. We are proposing a discussion within the surgical community to re-evaluate the extent of bony debridement.

**Take home message**

- In bony debridement of chronic osteomyelitis, wide, tumour-like or segmental resection and hunting for paprika sign in quest of removing all dead bone may not be necessary any more in all cases.

- Control of infection can be achieved with adequate thorough debridement, multidisciplinary team approach, and with high above minimum inhibitory concentration of local antibiotic.
